# Auxin efflux carrier *ZmPIN1a* modulates auxin reallocation involved in nitrate-mediated root formation

**DOI:** 10.1186/s12870-023-04087-0

**Published:** 2023-02-03

**Authors:** Yubin Wang, Jiapeng Xing, Jiachi Wan, Qingqing Yao, Yushi Zhang, Guohua Mi, Limei Chen, Zhaohu Li, Mingcai Zhang

**Affiliations:** 1 State Key Laboratory of Plant Physiology and Biochemistry, College of Agronomy and Biotechnology, China Agricultural University, Beijing, 100193 China; 2 Shandong Academy of Agricultural Sciences, Jinan, Shandong China; 3 College of Resources and Environmental Science, China Agricultural University, Beijing, 100193 China; 4 Center for Crop Functional Genomics and Molecular Breeding, China Agricultural University, Beijing, 100193 China

**Keywords:** Auxin, Auxin efflux carrier ZmPIN1a, Low nitrate, Maize, Root formation

## Abstract

**Background:**

Auxin plays a crucial role in nitrate (NO_3_^–^)-mediated root architecture, and it is still unclear that if NO_3_^–^ supply modulates auxin reallocation for regulating root formation in maize (*Zea mays L*.). This study was conducted to investigate the role of auxin efflux carrier ZmPIN1a in the root formation in response to NO_3_^–^ supply.

**Results:**

Low NO_3_^–^ (LN) promoted primary root (PR) elongation, while repressed the development of lateral root primordia (LRP) and total root length. LN modulated auxin levels and polar transport and regulated the expression of auxin-responsive and -signaling genes in roots. Moreover, LN up-regulated the expression level of *ZmPIN1a*, and overexpression of *ZmPIN1a* enhanced IAA efflux and accumulation in PR tip, while repressed IAA accumulation in LRP initiation zone, which consequently induced LN-mediated PR elongation and LR inhibition. The inhibition rate of PR length, LRP density and number of *ZmPIN1a*-OE plants was higher than that of wild-type plants after auxin transport inhibitor NPA treatment under NN and LN conditions, and the degree of inhibition of root growth in *ZmPIN1a*-OE plants was more obvious under LN condition.

**Conclusion:**

These findings suggest that *ZmPIN1a* was involved in modulating auxin levels and transport to alter NO_3_^–^-mediated root formation in maize.

**Supplementary Information:**

The online version contains supplementary material available at https://doi.org/10.1186/s12870-023-04087-0.

## Instruction

Nitrogen (N), a critical growth limiting nutrient, is an essential component of several biomolecules and plays a vital role in plant growth and development [1]. Besides, N fertilizers are widely used to ensure higher crop yields. In order to optimize N capture, plants often regularize root architecture to balance N status of the plant and soil [2]. Meanwhile, the heterogeneous reallocation and availability of N nutrients within the soil lead to the development of morphological and physiological plasticity of root architecture with specific functions [3, 4]. Among the various forms of N fertilizers, nitrate (NO_3_^–^) is the major form of available inorganic N utilized by most of the crop plants [5]. It has been demonstrated that the localized supply of NO_3_^–^ modulates the root length and lateral root (LR) formation [5, 6].

Apart from its nutritive role, NO_3_^–^ also acts as a signaling molecule modulating the root morphology to allow the plants to efficiently adapt to N supply [7, 8]. In Arabidopsis, NO_3_^–^ has a dual effect on the LR development depending on the concentration and localized supply of NO_3_^–^. For examples, low homogeneous NO_3_^–^ supply promotes LR elongation [9, 10], while represses the LR emergence [6]. Under heterogeneous N supply, high NO_3_^–^ stimulates the LR growth, but low NO_3_^–^ represses LR meristem activation [9, 11]. Unlike Arabidopsis, low NO_3_^–^ supply promotes the elongation of primary root (PR) and LR in maize, while reduces the density of lateral root primordia (LRP), which leads to decline in the overall length of LR [8, 12]. Similarly, N deficiency induces the root elongation and inhibit LR development in rice and wheat [13–15]. Although the knowledge of the NO_3_^–^-mediated root architecture is much documented, the understanding of the underlying integrated molecular and physiological mechanism remains inadequate.

Many studies have demonstrated that NO_3_^–^ stimulates the biosynthesis, polar transport, and signal transduction of auxin, which plays a vital role in the regulation of root development [16, 17]. Localized NO_3_^–^ supply does not induce the LR elongation in the auxin-insensitive mutant *Ataxr4*, which indicates a cross-talk between the auxin and NO_3_^–^ signaling pathways involved in NO_3_^–^-mediated root characteristics [18]. Besides, it has also been demonstrated that auxin biosynthesis gene *AtTAR2* is required for LR emergence in low NO_3_^–^-mediated root formation [16]. Meanwhile, low local NO_3_^–^ supply reduces auxin concentration in the phloem poles of the LR initiation zone led to less LRP, while enhances auxin accumulation in the root tip for longer roots [12, 17]. These suggested that NO_3_^–^-mediated root architecture relies on the distribution and accumulation of auxin in response to N supply.

It is well known that auxin polar transport plays a potential role in root development, which regulates auxin translocation to modulate the LR initiation and elongation [19, 20]. In Arabidopsis, there are eight AtPINs family members, which encode integral membrane proteins with conserved hydrophobic membrane-spanning domains and a less conserved central hydrophilic loop of variable length [21, 22]. In maize, there are eleven PINs genes, there members cluster within the AtPIN1 subclade, namely ZmPIN1a, ZmPIN1b, and ZmPIN1c. The ZmPIN1 proteins showed a typical structure similar to AtPIN1 [21–23]. AtPIN1 mainly resides at the basal end of the vascular cells, and *pin1* mutant display a slight reduction of root length and root meristem size [24]. In addition, OsPIN1 and OsPIN2 participate in auxin-dependent adventitious root emergence and LR formation [25, 26]. Correspondingly, the transcription of most auxin efflux carrier (e.g., AtPIN1, AtPIN2, AtPIN4, and AtPIN7) is controlled by NO_3_^–^ supply in Arabidopsis [27–29]. Localized partial NO_3_^–^ supply enhances the expression of auxin influx (OsAUX1 and OsAXR4) and efflux (OsPIN1c, OsPIN2, and OsPIN5b) carriers in rice, forming a robust auxin flux to LR zone for enhancing LR initiation [30, 31]. Besides, low NO_3_^–^ supply promotes root elongation and inhibits LR development via reduced auxin accumulation in the roots by down-regulation of *OsPINs* [13]. Recently, it has also been reported that OsPIN1b is involved in auxin transport to regulate root apical meristem activity for modulating the elongation of seminal roots under low NO_3_^–^ condition [14]. Taken together, these results demonstrate that polar auxin transport plays a vital role in NO_3_^–^-mediated root architecture.

Maize is a worldwide stable crop with the highest productivity among all crops; besides, it also demands the largest quantity of nitrogen-based fertilizers [32]. However, the global N use efficiency of maize is estimated around 25–50% [33], indicating the loss of more than half of the N fertilizer applied in maize production. Therefore, it is very imperative to explore the mechanisms involved in the root adaptation to N supply for improving N use efficiency in maize. As previously described, NO_3_^–^ -mediated root growth is regulated by auxin transport and allocation in maize roots subjected by heterogeneous or uniform NO_3_^–^ supply [12, 17]. Moreover, [17] documented that high NO_3_^–^ supply regulates the expression of *ZmPIN1a* and *ZmPIN9* involved in modulating early pericycle cell division and LR formation in shoot-borne roots by RNA-seq analysis. Moreover, *ZmPIN1a* overexpression increases auxin transport from shoot to root, and enhances root growth in maize [34]. However, it is yet to be determined whether ZmPIN1a modulate NO_3_^–^ -mediated root growth by regulating auxin polar transport and localization in maize.

This study was aimed at investigating the role of auxin efflux carrier ZmPIN1a in the development of PR and LR in response to NO_3_^–^ supply. There had been identified variations in auxin accumulation and reallocation under NN and LN conditions via Non-invasive Micro-test Technology (NMT), DR5rev::RFP marker line, and HPLC–ESI–MS/MS. Meanwhile, we used *ZmPIN1a* overexpressing transgenic lines, combined with exogenous addition of auxin transport inhibitor NPA to explore the potential role of ZmPIN1a in the NO_3_^–^-mediated root architecture. Hence, this study further demonstrated that ZmPIN1a was involved in auxin levels and transport to alter NO_3_^–^-mediated root formation in maize.

## Results

### LN supply enhanced the PR elongation and repressed the LR development

**Fig. 1:**
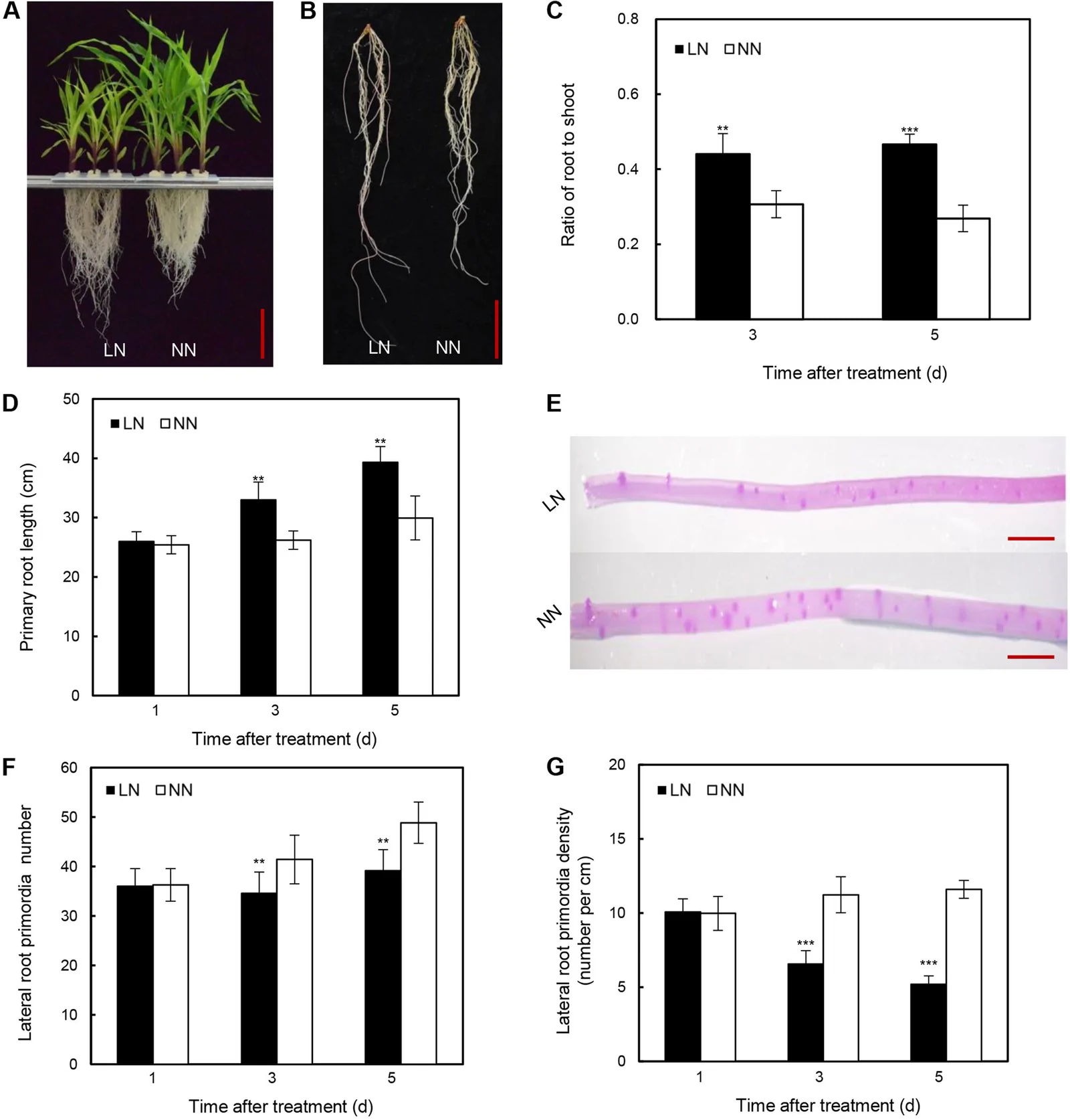
Morphology of maize seedlings under LN and NN conditions. **A** Phenotype characteristics of maize seedlings at 7 d under LN and NN conditions. Bars = 10 cm. **B** Representative root architecture system at 7 d under LN and NN conditions. Bars = 10 cm. **C** The ratio of root to shoot in maize seedlings at 3 and 5 d after LN treatment. **D** The PR length in maize seedlings at 1, 3, and 5 d after LN treatment. **E** Distribution of LRP in maize seedlings at 5 d after LN treatment. Bars = 3 mm. **F**-**G** The LRP number **F** and density **G** in maize seedlings at 1, 3, and 5 d after LN treatment. Values with error bars represent mean ± SD (*n* = 8). The asterisks indicated significant differences between different treatments according to a Student's t test. **, *P ≤* 0.01. PR, primary root; LRP, lateral root primordia; LN, low NO_3_^–^; NN, normal NO_3_^–^

Compared to NN, LN significantly inhibited the shoot growth while intensely promoted primary root elongation (Fig. 1A-B), and the root to shoot ratio was increased by respectively 52% and 70% at 3 and 5 d after LN treatment (Fig. 1C). Moreover, the PR length was increased by respectively 26% and 31% at 3 and 5 d after LN treatment (Fig. 1D). LN treatment repressed the development of LRP (Fig. 1E), and the number of LRP was reduced by respectively 16.5% and 20% at 3 and 5 d after LN treatment (Fig. 1F). The LRP density decreased by respectively 42% and 55% at 3 and 5 d after LN treatment (Fig. 1G). In addition, the total root length was decreased by respectively 10% and 31% at 3 and 5 d after LN treatment (Table S1). These results suggested LN significantly affected the root morphology in maize.

### LN supply modulated auxin levels in roots

**Fig. 2:**
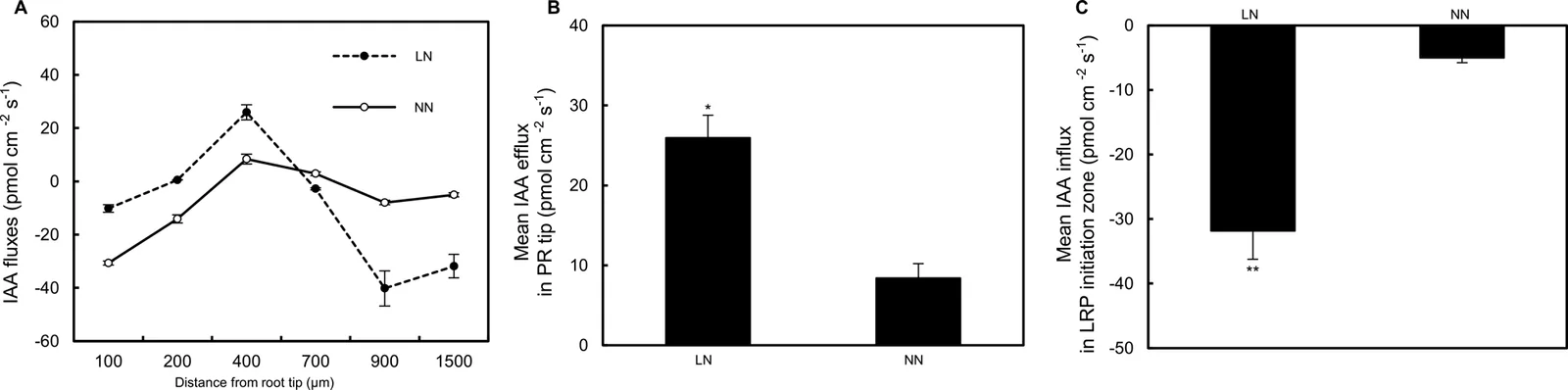
The IAA fluxes and content in PR tip and LRP initiation zone under LN and NN conditions. **A** IAA fluxes profile along the root apex of PR at 2 d after LN treatment. Each point represents the mean of three individual root. **B** Mean IAA efflux in PR tip zone (400 μm behind the root tip) at 2 d after LN treatment. **C** Mean IAA influx in LRP initiation zone (1500 μm behind the root tip) at 2 d after LN treatment. The asterisks indicated significant differences between different treatments according to a Student's t test. **, *P ≤* 0.01. PR, primary root; LRP, lateral root primordia

In order to evaluate the role of auxin in root formation exposed to different NO_3_^–^ supply, the IAA fluxes along the root tip were examined by using the NMT technique. As shown in Fig. 2A, the IAA fluxes showed the peak of the efflux in the meristem zone at 400 μM from the root tip, suggesting the higher levels of auxin in the meristem zone, which are consistent with previously published results on rice [35]. However, in the LRP initiation zone (500 to 1500 μM from root tip), the IAA fluxes were negative, which indicated a net IAA influx, suggesting the weak auxin activities in this region (Fig. 2A). Meanwhile, LN-treated plants had higher IAA flux values than NN-treated plants at 100, 200, and 400 μM from the root tip, while lower IAA flux values at 700, 900, and 1500 μM from the root tip. In addition, the mean IAA efflux in LN-treated plants was higher by 2.1 folds than that in NN-treated plants at 400 μM from the root tip (Fig. 2B). Inversely, the mean IAA influx in LN-treated plants was higher by 5.3 folds compared to NN-treated plants in the LRP initiation zone (at 1500 μM from the root tip) (Fig. 2C).

**Fig. 3:**
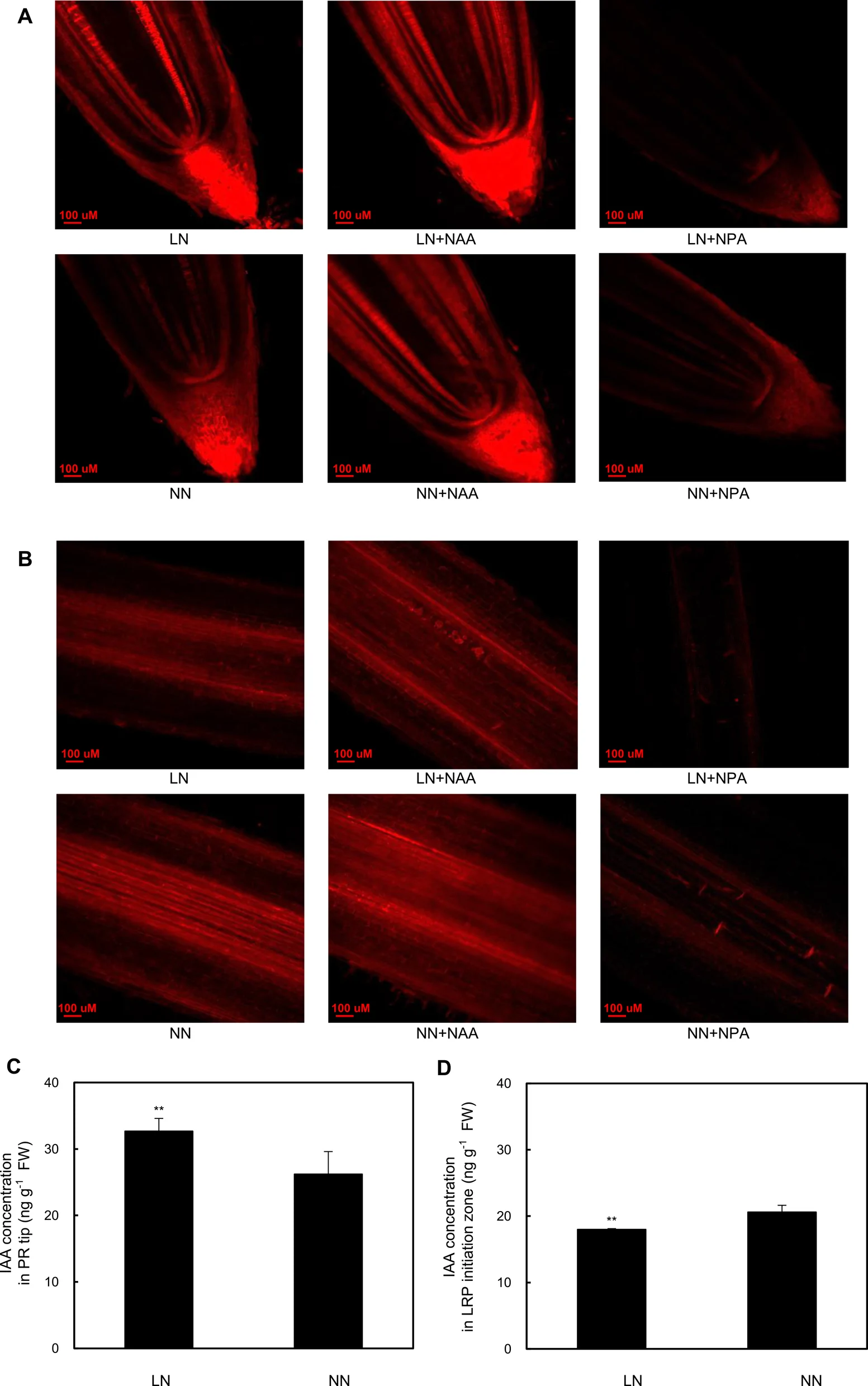
DR5rev::RFP signal and IAA content in PR tip and LRP initiation zone under LN and NN conditions. **A** DR5rev::RFP signal in PR tip (5 mm of root tip) at 2 d after LN treatment. Bars = 100 μm. **B** DR5rev::RFP signal in LRP initiation zone (5–25 mm behind the root tip) at 2 d after LN treatment. Bars = 100 μm. **C** IAA content in PR tip (5 mm of root tip) at 2 d after LN treatment. **D** IAA content in LRP initiation zone (5–25 mm behind the root tip) at 2 d after treatment. Values with error bars represent mean ± SD (*n* = 30). The asterisks indicated significant differences between different treatments according to a Student's t test. **, *P ≤* 0.01

To further assess auxin distribution and localization in maize roots responded to NO_3_^–^ supply, a DR5rev::RFP marker maize line was used to further evaluate IAA status of PR tip and LRP initiation subjected to alter N supply. As shown in Fig. 3A-B, application of naphthylacetic acid (NAA; an analogue of IAA) increased red fluorescent signal, while application of auxin transport inhibitor NPA decreased red fluorescent signal in roots under LN and NN conditions. Compared to NN, red fluorescent signal was stronger in PR tip (Fig. 3A), while weaker in LRP initiation zone under LN condition (Fig. 3B). Meanwhile, IAA levels in the PR tip and LRP initiation zone were measured by using HPLC–MS/MS method. Compared to NN, IAA levels increased by 25% in the PR tip (Fig. 3C), but decreased by 13% in the LRP initiation zone under LN condition (Fig. 3D). These results indicated that LN increased auxin accumulation in PR tip while decreased in the LRP initiation zone.

### LN supply regulated the expression of auxin-responsive and -signaling genes in roots

**Fig. 4:**
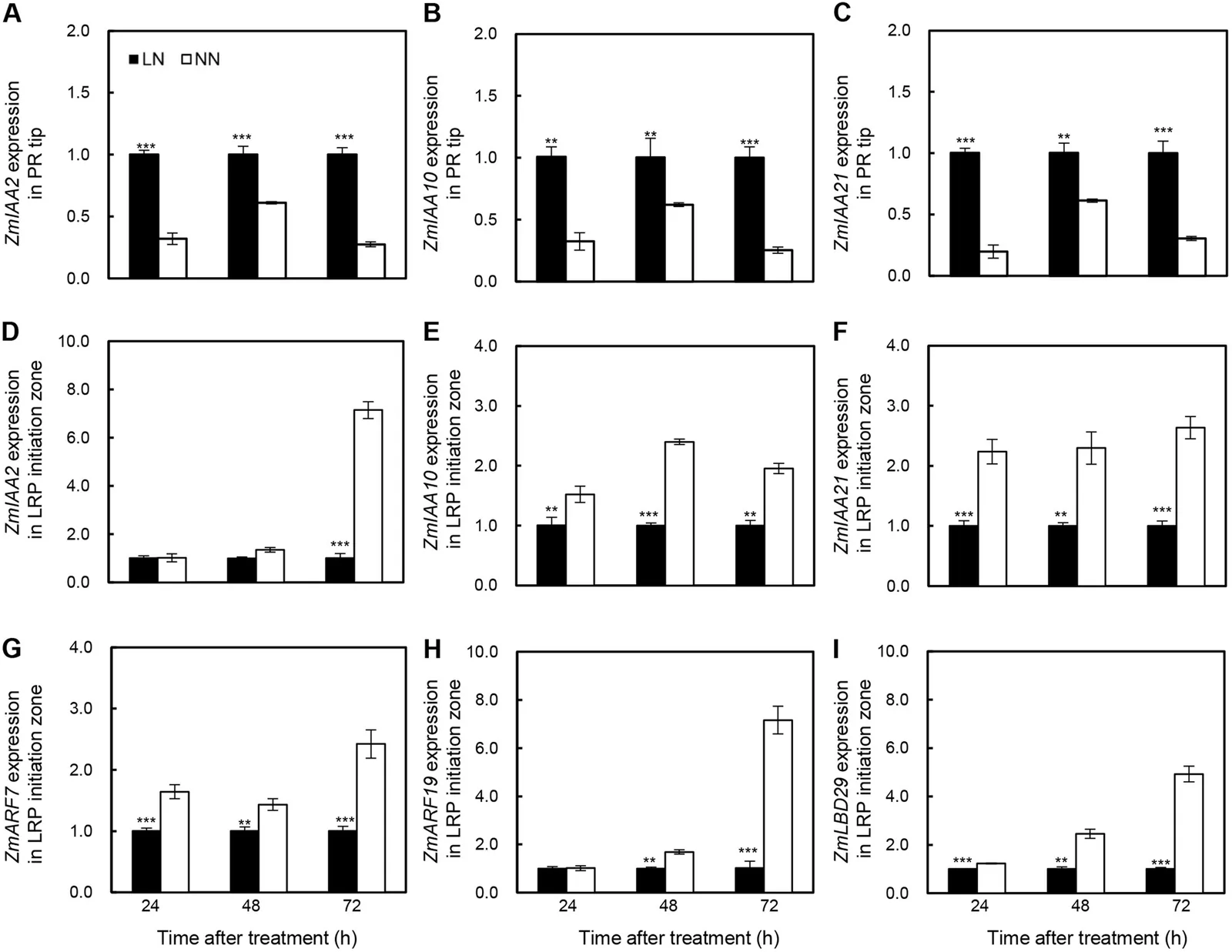
The relative expression level of auxin-response and signaling genes in PR tip and LRP initiation zone. **A**-**C** The relative expression level of auxin responsive genes *ZmIAA2* (**A**), *ZmIAA10* (**B**), and *ZmIAA21* (**C**) in PR tip at 24 h, 48 h, and 72 h after LN and NN treatments. **D**-**F** The relative expression level of auxin responsive genes *ZmIAA2* (**D**), *ZmIAA10* (**E**), and *ZmIAA21* (**F**) in LRP initiation zone at 24 h, 48 h, and 72 h after LN and NN treatments. **G**-**I** The relative expression level of auxin-signaling genes *ZmARF7* (**G**), *ZmARF19* (**H**), and *ZmLBD29* (**I**) in LRP initiation zone at 24 h, 48 h, and 72 h after LN and NN treatments. Values with error bars represent mean ± SD (*n* = 3). The gene expression was calibrated to the expression of *ZmUBC* (ubiquitin C). The asterisks indicated significant differences between different treatments according to a Student's t test. **, *P ≤* 0.01

To further confirm the changes of auxin levels induced the response of roots under LN and NN conditions, the transcription level of auxin-responsive genes (*ZmIAA2*, *ZmIAA10*, and *ZmIAA21*) were assessed using RT-qPCR. As expected, the relative expression levels of *ZmIAA2*, *ZmIAA10*, and *ZmIAA21* were significantly up-regulated in PR tip (Fig. 4A-C), while down-regulated in LRP initiation zone by LN (Fig. 4D-F). The genes (*ZmARF7*, *ZmARF19*, and *ZmLBD29*) involved in LR formation were also evaluated to assess the LRP formation response to LN, and the relative expression of these genes significantly down-regulated by LN in LRP initiation zone (Fig. 4G-I).

In addition, exogenous application of NAA increased the number and density of LRP under LN and NN conditions (Fig. S1). However, application of auxin transport inhibitor NPA decreased the number and density of LRP under LN and NN conditions (Fig. S2).

### LN supply altered the expression auxin transport gene *ZmPIN1a* in roots

**Fig. 5:**
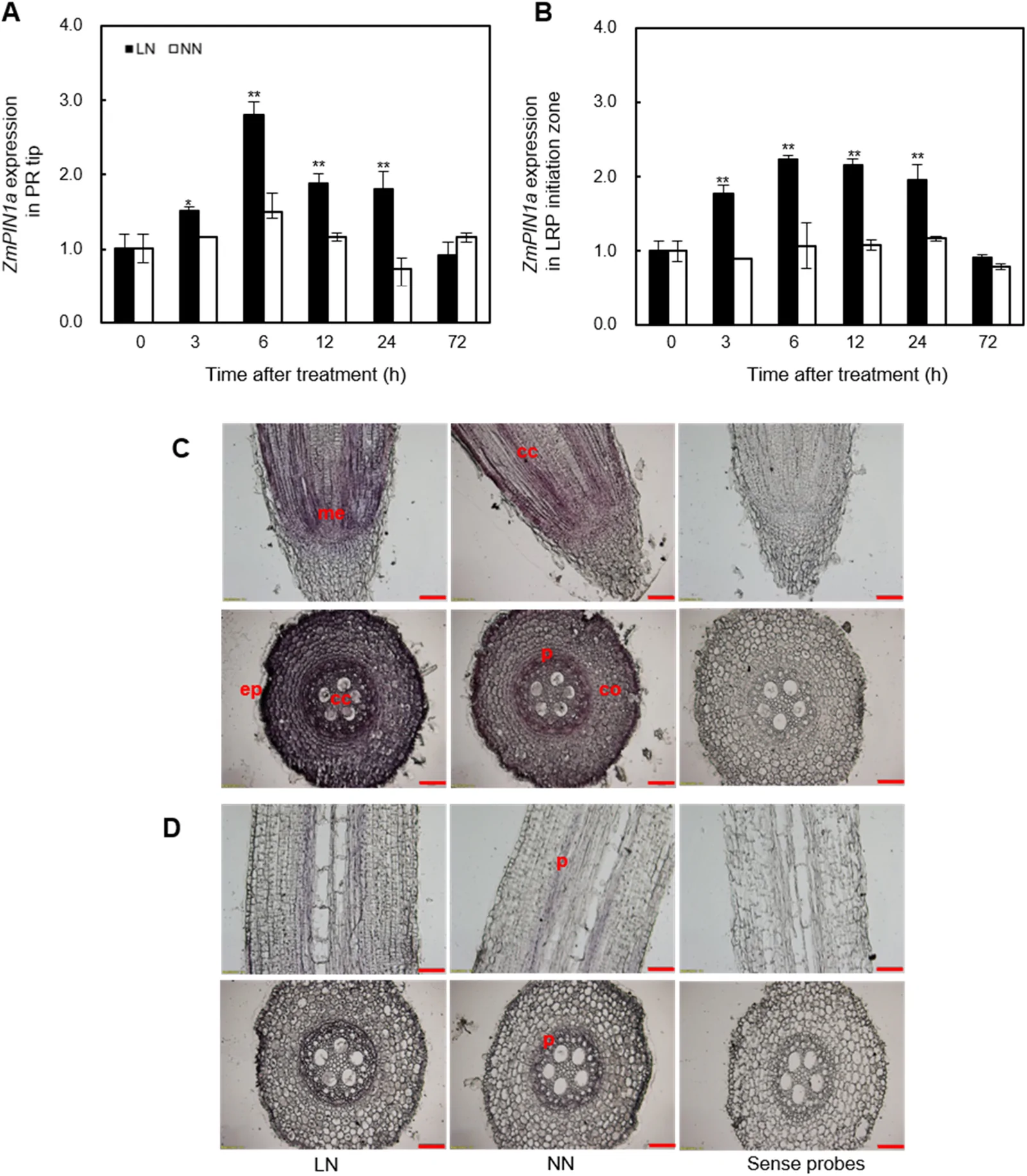
Effect of LN supply on the relative expression of *ZmPIN1a* in roots. **A**-**B** The relative expression of *ZmPIN1a* in PR tip (5 mm of the root tip) (**A**) and LRP initiation zone (5–25 mm behind the root tip) (**B**) at different time points after LN and NN treatments. Values with error bars represent mean ± SD (*n* = 3). The gene expression was calibrated to the expression of *ZmUBC* (ubiquitin C). **C**-**D** In-situ hybridization of *ZmPIN1a* in root at 12 h after LN and NN treatment. Longitudinal and transverse sections of maize PR tip (0–2 mm of the root tip) (**C**) and LRP initiation zone (7–10 mm behind the root tip) (**D**) probed with the *ZmPIN1a* antisense or sense probes. Bar = 100 µm. The asterisks indicated significant differences between different treatments according to a Student's t test. **, *P ≤* 0.01

Auxin polar transport is critical for accumulation and distribution of auxin in roots, which is carried by auxin influx and efflux transport proteins [20]. Previous study suggested that overexpression of *ZmPIN1a* increased auxin transport from shoot to root, and promoted root growth in maize [34]. Could ZmPIN1a be involved in modulating root formation under LN condition? The relative expression of *ZmPIN1a* was examined in the PR tip and LRP initiation zone at different times points after LN treatment. Compared to NN, the relative expression level of *ZmPIN1a* significantly up-regulated in PR tip (Fig. 5A), similar changes were observed in LRP initiation zone (Fig. 5B), where LN also markedly enhanced the transcript levels of *ZmPIN1a*. The expression patterns of *ZmPIN1a* in PR tip and LRP initiation zone were analysed at tissue level using in-situ hybridization. The red hybridization signal of *ZmPIN1a* was predominantly detected in the meristematic area, epidermis, and central cylinder of the PR tip (Fig. 5C). The expression levels of *ZmPIN1a* were significantly stimulated by LN treatment in the longitudinal and transverse of PR tip (Fig. 5C), while were also slightly stimulated by LN treatment in the longitudinal and transverse of LRP initiation zone (Fig. 5D). Therefore, ZmPIN1a might be involved in modulating root architecture under LN condition.

### Manipulating ZmPIN1a modified root architecture under LN condition

**Fig. 6:**
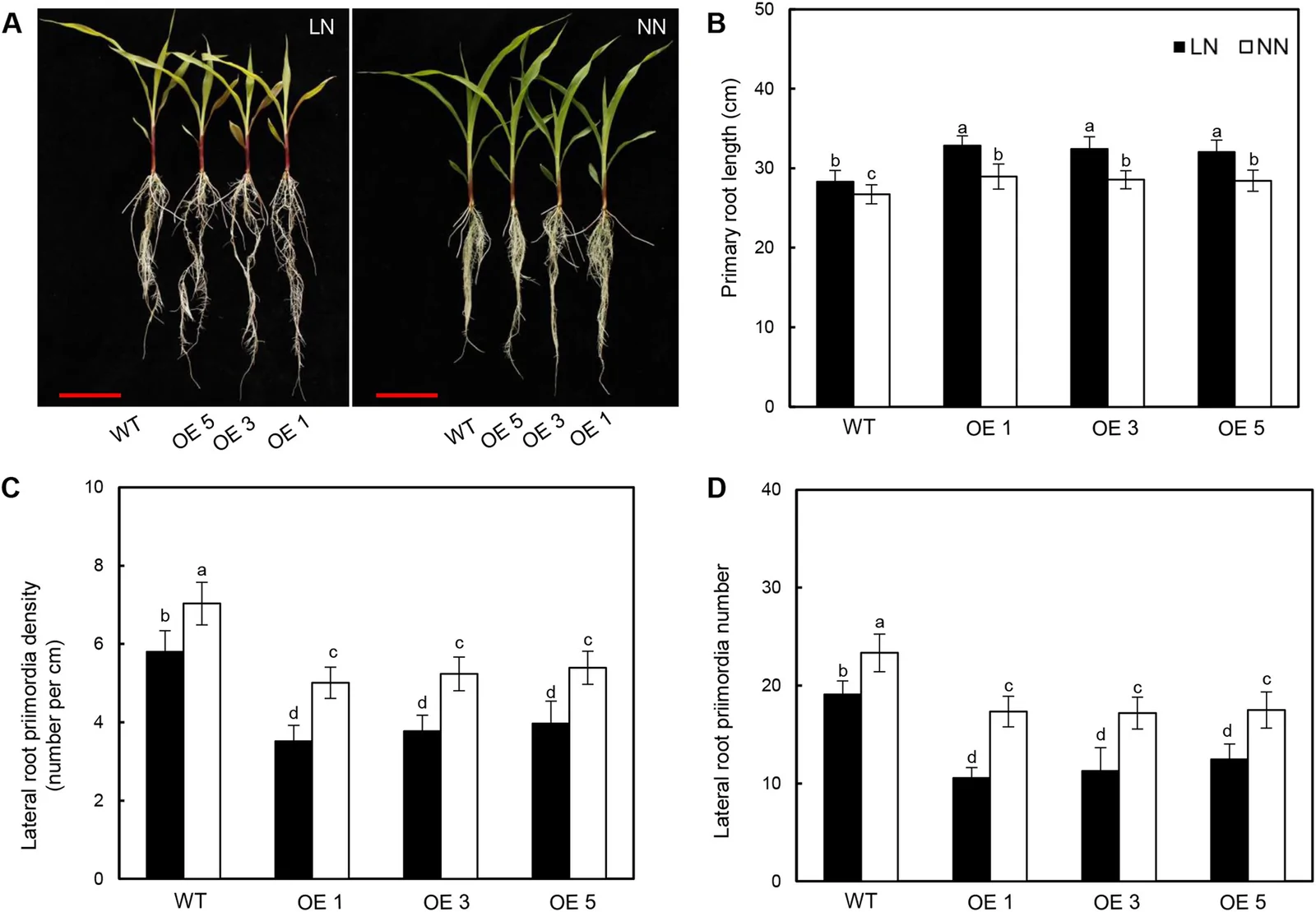
Phenotype characteristics of *ZmPIN1a*-OE plants subjected to LN or NN treatment. **A** Phenotype characteristics of *ZmPIN1a*-OE plants (OE 1, OE 3, and OE 5) at 7 d after LN and NN treatments. Bar = 10 cm. **B**-**D** The PR length (**B**), LRP density (**C**), and LRP number (**D**) in WT and *ZmPIN1a*-OE plants at 7 d after LN treatment. Values with error bars represent mean ± SD (*n* = 12). Different letters indicated a significant difference between different treatments calculated by Fisher's LSD (*P ≤* 0.05). WT, wild-type

In order to assess the role of ZmPIN1a in modulating root architecture, the transgenic lines overexpressing *ZmPIN1a* (*ZmPIN1a*-OE) were evaluated for root phenotypic characteristics under LN and NN conditions. *ZmPIN1a*-OE plants exhibited higher expression levels of *ZmPIN1a* (Fig. S3). Overexpression of *ZmPIN1a* significantly stimulated the PR elongation, while repressed the shoot growth related to the wild-type plants in response to LN and NN treatments (Fig. 6A). In comparison with wild-type plants, the PR length of *ZmPIN1a*-OE lines (OE 1, OE 3, and OE 5) increased by respectively 13.8, 12.7, and 11.7% under LN condition, but by 7.7, 6.3, and 5.9% under NN condition (Fig. 6B). The LRP density of *ZmPIN1a*-OE lines decreased by respectively 44.7, 40, and 34.6% under LN condition, but by 28.8, 25.5, and 23.4% under NN condition (Fig. 6C). And similar observations were presented in the number of LRP of *ZmPIN1a*-OE plants (Fig. 6D). These results suggested ZmPIN1a might be involved in LN-mediated root formation.

### Overexpression of ***ZmPIN1a*** changed auxin levels and transport in roots subjected to NO_3_^–^ supply

**Fig. 7:**
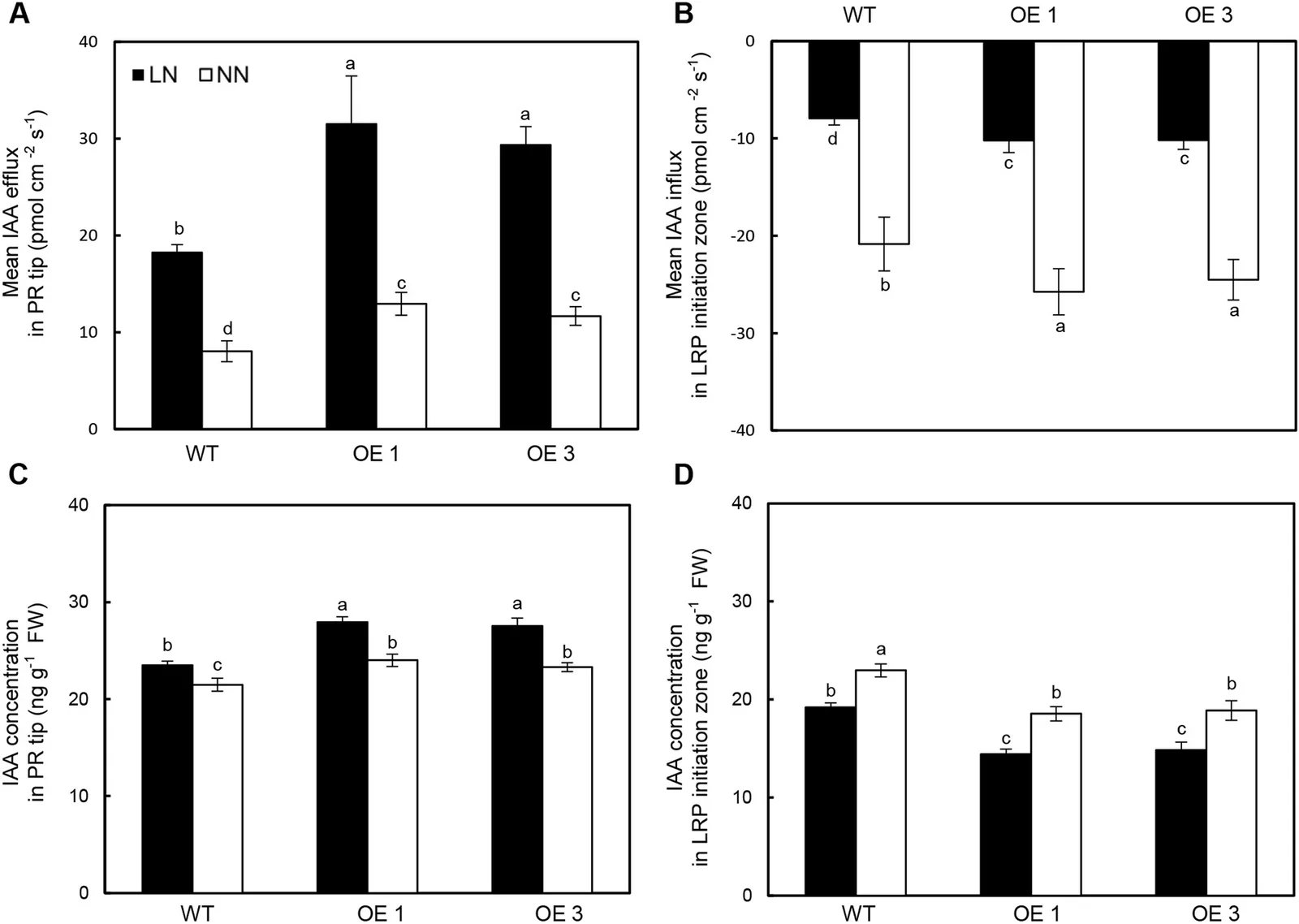
IAA fluxes and contents in PR tip and LRP initiation zone of *ZmPIN1a*-OE plants. **A**-**B** Mean IAA efflux in PR tip (**A**) and mean IAA influx in LRP initiation zone (**B**) of WT and *ZmPIN1a*-OE plants at 2 d after LN and NN treatments. **C**-**D** IAA content in PR tip (**C**) and LRP initiation zone (**D**) of WT and *ZmPIN1a*-OE plants at 2 d after LN and NN treatments. Values with error bars represent mean ± SD (*n* = 3). Different letters indicated a significant difference between different treatments calculated by Fisher's LSD (*P ≤* 0.05)

To evaluate the role of ZmPIN1a in modulating the auxin reallocation in roots under LN and NN conditions, IAA fluxes were examined in *ZmPIN1a*-OE roots by using the NMT technique. IAA effluxes in PR tip of *ZmPIN1a*-OE plants were higher by respectively 73 and 61% than those in wild-type plants under LN condition, but by 60 and 45% under NN condition (Fig. 7A). Meanwhile, IAA influxes in LRP initiation zone of *ZmPIN1a*-OE plants were higher by respectively 28.4 and 28.1% than those in the wild-type plants under LN condition, but by 23.5 and 17.5% under NN condition (Fig. 7B). The contents of IAA in PR tip and LRP initiation zone were also measured in the *ZmPIN1a*-OE plants. In comparison with wild-type plants, IAA contents in PR tip of *ZmPIN1a*-OE plants increased by respectively 19.0 and 17.2% under LN condition, but by 11.7 and 8.5% under NN condition (Fig. 7C). IAA contents in LRP initiation zone of *ZmPIN1a*-OE plants decreased by respectively 25.0 and 22.8% under LN condition, but by 19.2 and 17.8% under NN condition (Fig. 7D).

**Fig. 8:**
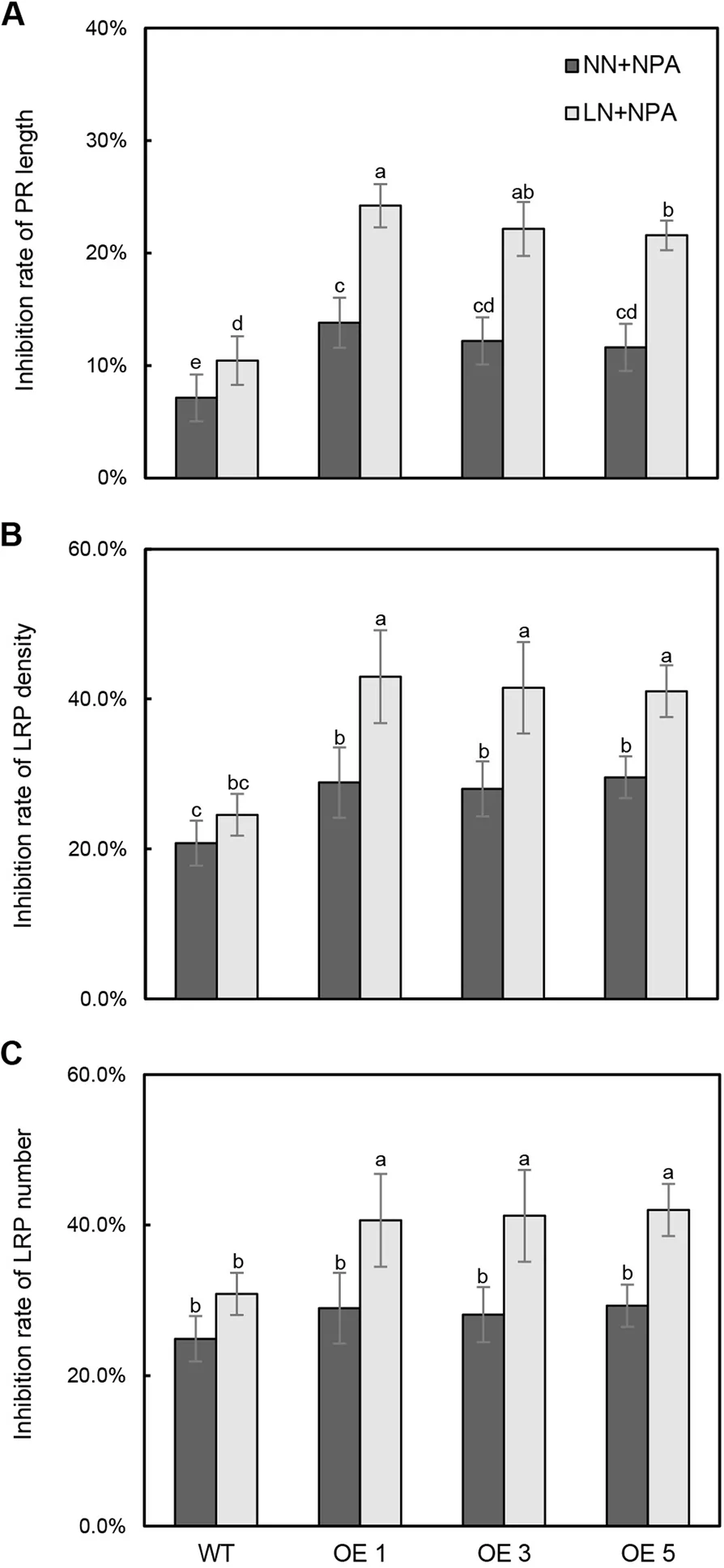
Effects of auxin transport inhibitor NPA on PR and LRP of *ZmPIN1a*-OE plants. **A**-**C** The inhibition rate of PR length (**A**), of LRP density (**B**), and LRP number (**C**) of *ZmPIN1a*-OE plants after NPA treatment under NN and LN conditions. Inhibition rate = [(NN + NPA)—NN] /NN, [(LN + NPA)—LN] /LN. Values with error bars represent mean ± SD (*n* = 8). Different letters indicated a significant difference between different treatments calculated by Fisher's LSD (*P ≤* 0.05)

In addition, when auxin transport inhibitor NPA was applied under NN condition, the inhibition rate of PR length, LRP density and number of *ZmPIN1a*-OE plants was higher than that of wild-type plants. More importantly, with the application of NPA under LN condition, the inhibition rate of PR length, LRP density and number of *ZmPIN1a*-OE plants significantly increased (Fig. 8A-C). These results suggested ZmPIN1a could influence auxin levels and polar transport from shoot to root to alter root formation under LN condition.

## Discussion

Modification in the root architecture has been recognized as an essential adaptive strategy for plants to obtain nutrients from the soil. Previous studies have shown that localized NO_3_^–^ supply affects the root growth and development in diverse species, which modulate N uptake [9, 17, 30]. Here, uniform LN significantly repressed the LR formation while promoted the PR elongation (Fig. 1), which decreased total root length (Table S1). Although LRs are the major contributors to N uptake, the axial roots could play a more important role for trapping NO_3_^–^ from the deeper soil under LN than NN [8].

Many studies have demonstrated that auxin transport and allocation are involved in N-modulated root formation in diverse species [16, 17]. Whether NO_3_^–^ treatment affects the auxin transport along the roots has yet to be fully uncovered in maize. Here, we provide evidence that auxin transport is crucial for NO_3_^−^-mediated root formation. Previous studies have shown that modification of IAA flux affects IAA content, and finally regulate plant development [35, 36]. In this study, the maximal IAA efflux occurred in the meristem zone of the maize root, and similar results are observed in rice [35], which indicated that higher levels of auxin are present in the meristem zone. Moreover, LN increased the IAA efflux in the meristem zone of root tip, while increased the IAA influx in the LRP initiation zone (Fig. 2). This finding corresponds to the results of uniform LN treatment enhanced the IAA levels in the PR tip, while reduced the IAA levels in LRP initiation zone (Fig. 3C-D). These results demonstrated that NO_3_^−^ exerts an effect on IAA flux in an indirect manner, which could result in auxin level change in maize root.

In this study, the auxin-responsive marker DR5rev::RFP revealed a more detailed topological view of the effect of LN treatment on the auxin response in PR tip and LRP initiation zone. In the LN-treated plants, the PR tip displayed stronger signal of the auxin-responsive marker DR5rev::RFP (Fig. 3A), responding to the higher IAA effluxes (Fig. 2B) and contents (Fig. 3C), which was consistent with the increased expression of auxin-responsive genes including *ZmIAA2*, *ZmIAA10*, and *ZmIAA21* (Fig. 4A-C). Similar findings are observed in maize and wheat, where aluminum stress reduces IAA levels and down-regulates the auxin-responsive genes including *IAA2*, *IAA10*, and *IAA21* in PRs for repressing root elongation [37, 38]. Under LN condition, we found that the expression levels of *ZmIAA2*, *ZmIAA10*, and *ZmIAA21* were lower (Fig. 4D-F), IAA influxes were higher, IAA contents were lower, and the signal of auxin-responsive marker DR5rev::RFP were weaker in LRP initiation zone than under NN condition. In addition, the LRP initiation is under the control of auxin-responsive transcription factors *ARF7* and *ARF19*, which directly target the downstream auxin-mediated transcription of lateral organ bounda-ries-domain29/asymmetric leaves2-like16 (LBD29) in roots, both of which positively regulate LRP formation [39–41]. Here, the expression of *ZmARF7* and *ZmARF19*, along with their target gene *ZmLBD29* were significantly down-regulated by LN treatment in the LRP initiation zone (Fig. 4G-I), consistent with the result of LN inhibiting the LRP formation. In addition, NAA application increased the density of LRP, while NPA treatment reduced the same under LN as well as NN conditions (Fig. S1-2). Similar findings are also observed in previous studies [13, 16, 42]. These results further demonstrated that NO_3_^−^ exerts an effect on IAA flux in root tips and LRP initiation zone, resulting in a higher IAA content in PR tips, and a longer PR length, a lower IAA content in LRP initiation zones, and a lower LRP density. Consequently, it was inferred that auxin distribution and local concentration variation played important roles to alter auxin signaling for modulating root elongation and LRP formation under different N conditions.

Auxin is known as the focal point of environmental and endogenous signal perception for root formation via regulating auxin polar transport [43]. The polarity of auxin transport is altered by the PIN, AUX1/LAX and ABCB gene families [12, 17, 23, 44] Auxin efflux carrier PIN1 has been identified in *Arabidopsis*, rice, and maize [23]. The *pin1* mutant in both rice and Arabidopsis display a reduction of root length [14, 24]. Similar to the Arabidopsis AtPIN1 orthologue, which controls auxin transport from shoot to root, *ZmPIN1a* was also found to be involved in regulating plant root development [34]. However, the role of ZmPIN1a in NO_3_^–^-mediated root growth was not observed in maize. Here, *ZmPIN1a*-OE plants were used to investigate the *ZmPIN1a* on root formation in response to NO_3_^–^ supply. Our results showed that ZmPIN1a was predominantly expressed in the meristematic area, epidermis, and central cylinder of the PRs, and LN significantly up-regulated the expression of *ZmPIN1a* in PR tip and LRP initiation zone, (Fig. 5). Considering the increased *ZmPIN1a* expression increased auxin accumulated in root tips [34], and LN treatment enhanced IAA levels in PR tips (Fig. 2). We assumed that ZmPIN1a modulates auxin reallocation involved in nitrate-mediated root formation. Here, *ZmPIN1a*-OE plants had higher IAA efflux in root tip and influx in LRP initiation zone under LN and NN conditions (Fig. 7A-B). Correspondingly, overexpression of *ZmPIN1a* enhanced IAA accumulation in root tip, while decreased IAA levels in the LRP initiation zone (Fig. 7C-D). These findings suggested that LN treatment affected the expression of *ZmPIN1a* gene, the expression of *ZmPIN1a* gene promoted the net auxin flux and auxin transport, further leaded to an evident enhancement of IAA levels in root tips.

Furthermore, overexpression of *ZmPIN1a* enhanced PR elongation and repressed LR formation, and the PR elongation and LR formation in *ZmPIN1a*-OE plants exhibited a higher degree of sensitivity under LN condition (Fig. 6). More importantly, the inhibition of PR length of *ZmPIN1a*-OE plants was significantly higher than that of wild-type by NPA, and the inhibition rate was significantly higher under LN condition than that under NN condition, and similar observations were presented in LRP density and number of *ZmPIN1a*-OE plants (Fig. 8A-C). These indicated that ZmPIN1a was essential to modulate auxin levels for building the root architecture in response to N availability. The interpretation of these observations was that *ZmPIN1a* acted to drain auxin from the LRP initiation zone to root tips, resulting in the auxin level accumulation in root tips under LN condition. However, in the LRP initiation zone, auxin falling below a threshold sufficient to support normal LRP initiation. This result provided further evidence about the role of auxin polar transport in NO_3_^–^-mediated root growth in maize. How ZmPIN1a regulated auxin transport from LRP initiation to root tips in the process of LN-mediated root development needs to be explored in future.

## Conclusions

Taken together, our results suggested that the auxin efflux carriers ZmPIN1a played an important role in LN-mediated PR elongation coupled with LR inhibition via altering the auxin levels and transport in root tip and LRP initiation zone. Our studies provide valuable knowledge that would pave the way for expediting crop breeding programs involving genetic improvement in N use efficiency through root architecture modifications.

## Materials and methods

### Plant materials and growth conditions

*ZmPIN1a*-overexpressing plants (OE 1, OE 3, and OE 5) were obtained from Crop Functional Genomics and Molecular Breeding Research Center of China Agricultural University. The lines were produced in maize inbred line B73-329 background. For hydroponics, seeds of the maize varieties ZD958, B73-329 and transgenic lines were surface sterilized in 10% (v/v) H_2_O_2_ for 20 min, rinsed with distilled water five times and then sown in the sand. Seven days after germination, the endosperm of each seeding was excised, and uniform seedings with two visible leaves were transferred to a hydroponic box containing 5 L nutrient solution. The experiment was conducted in a growth chamber with 16-h-light (28 °C)/8-h-dark (22 °C) and the relative humidity controlled to approximately 70 to 80%. The basic nutrient solution contained 2 mM KNO_3_, 1 mM CaCl_2_, 0.1 mM KH_2_PO_4_, 0.5 mM MgSO_4_, 0.1 mM EDTA-Fe, 0.03 mM H_3_BO_3_, 0.0025 mM ZnSO_4_, 0.008 mM CuSO_4_, 0.005 mM MnSO_4_ and 0.0003 mM (NH_4_)_6_Mo_7_O_24_. Plants were supplied with half-strength nutrient solution for 2 d and then transferred into a solution with either low NO_3_^–^ (LN, 0.05 mM) or normal NO_3_^–^ (NN, 2 mM) as control. To exclude the possibility of potassium (K^+^) interference, the concentrations of K^+^ in the LN solution were supplemented to the same levels as those of the NN solution using KCl. For all growth solutions, the pH was adjusted to 5.8–6.0 with KOH. The nutrient solution was renewed every other day and aerated continuously by an electric pump.

### Measurement of root morphology

The seedlings were sampled after N treatment. The PR length was measured with a ruler. The total root length was measured with the scanner (Epson V700, Beijing, China) and the images were analysed using WinRHIZO Pro 5.0 (Quebec City, Canada). Feulgen staining was used to estimate the number and density of lateral root primordium (LRP) in the apical unbranched zone of the PR.

### Measurement of IAA flux in roots

Indole-3-acetic acid (IAA) flux in roots was measured using Non-invasive Micro-test Technology (NMT) system (Younger USA LLC, Amherst, MA 01,002, USA; youngeru-sa.com) by Xuyue (Beijing) Sci. & Tech. Co., Ltd., Beijing, China, as described previously [35, 36]. For each experimental group, 3 uniform seedlings were selected at 2 d after LN treatment, and the roots were immediately equilibrated for 15 min in the test buffer (0.05/2 mM KNO_3_, 0.1 mM KCl, 0.1 mM CaCl_2_, 0.1 mM MgCl_2_, 0.3 mM MES, pH 6.0). The IAA fluxes were determined at concentrating on the following zone including 100, 200, 400, 700, 1500 and 2000 μm from the root tip, and then the IAA flux was measured at 400 and 1500 μm from the root tip. IAA fluxes were recorded continuously for 3 min to yield a steady mean value. At least three individual plants were measured for each treatment. The positive and negative values indicated net IAA efflux and influx, respectively.

### Microscopic analyses

In order to observe the auxin signaling response to LN treatment in the roots, the transgenic line carrying DR5rev::RFP auxin response marker with a synthetic auxin promoter [45] was used. Following the exposure of DR5rev::RFP seedlings to LN treatment for 24 h, the root tip were subsequently scanned for the RFP signal using Zeiss LSM 880.

### Determination of IAA concentration

Two segments were collected from the roots at 2 d after LN treatment. The first segment (0–5 mm root tip) was as the root meristem controlling root growth, and other segment (5–25 mm behind the root tip of the PR) was the LRP initiation and development zone. The free IAA was quantified using high-performance liquid chromatog-raphy-electrospray ionization tandem mass spectrometry (HPLC–ESI–MS/MS). The root samples were ground to fine powder in liquid nitrogen and each sample (50 mg) was transferred to 1.5 mL tubes and added 50 µL of the working solution of internal standards (2H-labeled IAA). Subsequently, 500 µL of extraction solvent (2-propanol/H_2_O/concentrated HCl, 2:1:0.002, v/v/v) was added to each sample and vortexed. The remaining steps were performed as previously reported by [46].

### RNA isolation and quantitative *RT-PCR* (*qRT-PCR*) analysis

Total RNA was extracted from the roots using the Plant RNeasy Mini kit (Tiangen). For RT-qPCR analysis, 1ug total RNA was reverse transcribed into cDNA using Oligo d (T) 18 primer and M-MLV reverse transcriptase (Takara, Japan). The diluted cDNA was amplified using SYBR Premix EX TaqTM (Takara, Japan) on an Applied Biosystems 7500 Fast Real-Time PCR System (Applied Biosystems, USA) following the manufacturer’s protocol. *ZmUBC* (ubiquitin C) was used to normalize gene expression. Fold change of gene expression values were calculated using the 2^−ΔΔCT^ methods [47]. All primers were listed in Table S2.

### In-situ hybrid dization

In-situ hybridization experiments have been described previously [48] with some modifications. The PR tip were harvested at 12 h after LN treatment, fixed in 3.7% for-mal-acetic-alcohol, vacuum filtered for 15 min, and fixed overnight in a fresh fixative solution. The samples were dehydrated with a graded series of ethanol and xylene, and finally embedded in paraffin. The 10 µm-thick root sections obtained using Microtome (Leica Germany) were transferred to ice-cold DEPC water. Digoxigenin-labeled probes were synthesized for cDNA using gene-specific primers, including SP6 and T7 RNA polymerase binding sites. The sense and antisense probes were generated using DIG RNA Labeling Kit (SP6/T7, Roche). The sense probe was produced with SP6 RNA polymerase as negative control. Other steps were performed as previously reported [44]. The details of the primers used for probe synthesis are given in Table S2.

### Nutrient solution treatments with NAA and NPA

Maize ZD958 seedlings were treated with naphthylacetic acid (NAA; an analogue of IAA) and auxin-transport inhibitor NPA when the nutrient solution was changed every time. The final concentration of NAA was 1 μM, NPA was 0.1 μM. NAA and NPA were first dissolved in dimethyl sulfoxide (DMSO). The same dose of DMSO was added to the control.

### Lanolin paste treatment of NPA

For the experiment of *ZmPIN1a*-OE plants applied with NPA treatment, ZmPIN1a-OE plants were grown in half-strength nutrient solution for 2 d, the lanolin paste containing NPA was smeared uniformly surround the hypocotyl of plants when the nutrient solution was changed every time. The concentration of NPA was 1000 μM. The control plants were treated with lanolin paste without NPA. The method was performed as previously reported by [49].

## Statistical analysis

The data were statistically analysed using the SAS 9.0 (SAS Institute Ltd., USA). The student’s test was used for the comparisons between two groups of date. For the data sets of more than two groups, one-way ANOVA with LSD (*P ≤* 0.05) was used.

## Supplementary Information

**Additional file 1:Fig. S1.** Effects of auxin on root growth under LN and NN conditions. **Fig. S2.** Effects of auxin inhibitor NPA on root growth under LN and NN conditions. **Fig. S3.** Relative expression level of *ZmPIN1a* in roots of *ZmPIN1a*-OE plants. **Table S1.** Effects of LN supply on total root length. **Table S2.** List of primers used in this study.

## Data Availability

All data generated or analyzed during this study are available from the corresponding author on reasonable request.

## References

[1] Hawkesford M. The diversity of nitrogen use efficiency for wheat varieties and the potential for crop improvement. Better Crops. 2012;96:10–2. 10.1007/s11103-018-0812-z.

[2] O’Brien J, Vega A, Bouguyon E, Krouk G, Gojon A, Coruzzi G, Gutierrez RA. Nitrate transport, sensing, and responses in plants. Mol Plant. 2016;9:837–56. 10.1016/j.molp.2016.05.004. 27212387

[3] García MJ, Romera FJ, Lucena C, Alcántara E, Pérez-vicente R. Ethylene and the regulation of physiological and morphological responses to nutrient deficiencies. Plant Physiol. 2015;169:51–60. 10.1104/pp.15.00708. 26175512 PMC4577413

[4] Shahzad Z, Amtmann A. Food for thought: how nutrients regulate root system architecture. Curr Opin Plant Biol. 2017;39:80–7. 10.1016/j.pbi.2017.06.008. 28672167 PMC5605224

[5] Nacry P, Bouguyon E, Gojon A. Nitrogen acquisition by roots: physiological and developmental mechanisms ensuring plant adaptation to a fluctuating resource. Plant Soil. 2013;370:1–29. 10.1007/s11104-013-1645-9.

[6] Zhang HM, Rong HL, Pilbeam D. Signalling mechanisms underlying the morphological responses of the root system to nitrogen in Arabidopsis thaliana. J Exp Bot. 2007;58:2329–38. 10.1093/jxb/erm114. 17578866

[7] Krouk G, Ruffel S, Gutierrez RA, Gojon A, Crawford NM, Coruzzi GM, Lacombe B. A framework integrating plant growth with hormones and nutrients. Trends Plant Sci. 2011;16:178–82. 10.1016/j.tplants.2011.02.004. 21393048

[8] Gao K, Chen FJ, Yuan LX, Zhang FS, Mi GH. A comprehensive analysis of root morphological changes and nitrogen allocation in maize in response to low nitrogen stress. Plant, Cell Environ. 2015;38:740–50. 10.1111/pce.12439. 25159094

[9] Zhang H, Forde BG. An Arabidopsis MADS box gene that controls nutrient-induced changes in root architecture. Science. 1998;279:407–9. 10.1126/science.279.5349.407. 9430595

[10] Tian QY, Sun P, Zhang WH. Ethylene is involved in nitrate-dependent root growth and branching in Arabidopsis thaliana. New Phytol. 2009;184:918–31. 10.1111/j.1469-8137.2009.03004.x. 19732351

[11] Ruffel S, Poitout A, Krouk G, Coruzzi GM, Lacombe B. Long-distance nitrate signaling displays cytokinin dependent and independent branches. J Integr Plant Biol. 2016;58:226–9. 10.1111/jipb.12453. 26619818

[12] Tian QY, Chen FJ, Liu JX, Zhang FS, Mi GH. Inhibition of maize root growth by high nitrate supply is correlated with reduced IAA levels in roots. J Plant Physiol. 2008;165:942–51. 10.1016/j.jplph.2007.02.011. 17928098

[13] Sun HW, Tao JY, Liu SJ, Huang SJ, Chen S, Xie XN, Yoneyama KC, Zhang YL, Xu GH. Strigolactones are involved in phosphate- and nitrate-deficiency-induced root development and auxin transport in rice. J Exp Bot. 2014;65:6735–46. 10.1093/jxb/eru029. 24596173 PMC4246174

[14] Sun HW, Tao JY, Bi Y, Hou MM, Lou JJ, Chen XN, Zhang XH, Luo L, Xie XN, Yoneyama K, Zhao QZ, Xu GH. OsPIN1b is involved in rice seminal root elongation by regulating root apical meristem activity in response to low nitrogen and phosphate. Sci Rep. 2018;8:1–11. 10.1038/s41598-018-29784-x. 30158652 PMC6115472

[15] Xu YH, Ren YZ, Li JJ, Li L, Chen SL, Wang ZQ, Xin ZY, Chen F, Lin TB, Cui DQ, Tong YP. Comparative proteomic analysis provides new insights into low nitrogen-promoted primary root growth in hexaploid wheat. Front Plant Sci. 2019;10:151. 10.3389/fpls.2019.00151. 30842781 PMC6391680

[16] Ma WY, Li JJ, Qu BY, He X, Zhao XQ, Li B, Fu XD, Tong YP. Auxin biosynthetic gene TAR2 is involved in low nitro-gen-mediated reprogramming of root architecture in Arabidopsis. Plant J. 2014;78:70–9. 10.1111/tpj.12448. 24460551

[17] Yu P, Eggert K, von Wirén N, Li CJ, Hochholdinger F. Cell type-specific gene expression analyses by RNA sequencing reveal local high nitrate-triggered lateral root initiation in shoot-borne roots of maize by modulating auxin-related cell cycle regulation. Plant Physiol. 2015;169:690–704. 10.1104/pp.15.00888. 26198256 PMC4577424

[18] Zhang HM, Jennings A, Barlow PW, Forde BG. Dual pathways for regulation of root branching by nitrate. Proc Natl Acad Sci USA. 1999;96:6529–34. 10.1073/pnas.96.11.6529. 10339622 PMC26916

[19] Benková E, Michniewicz M, Sauer M, Teichmann T, Seifertová D, Jurgens G, Friml J. Local, Efflux-dependent a module for plant organ formation. Cell. 2003;115:591–602. 10.1016/S0092-8674(03)00924-3. 14651850

[20] Petrasek J, Friml J. Auxin transport routes in plant development. Development. 2009;136:2675–88. 10.1242/dev.030353. 19633168

[21] Forestan C, Farinati S, Varotoo S. The maize PIN gene family of auxin transporters. Front Plant Sci. 2012;3:16. 10.3389/fpls.2012.00016. 22639639 PMC3355596

[22] Zwiewka M, Bilanovičová V, Seifu YW, Nodzyński T. The Nuts and Bolts of PIN auxin efflux carriers. Front Plant Sci. 2019;10:985. 10.3389/fpls.2019.00985. 31417597 PMC6685051

[23] Matthes M, Best N, Robil J, Malcomber S, Gallavotti A, Mcsteen P. Auxin EvoDevo: conservation and diversification of genes regulating auxin biosynthesis, transport, and signaling. Mol Plant. 2019;12:298–320. 10.1016/S0168-9452(97)00151-9. 30590136

[24] Blilou I, Xu J, Wildwater M, Willemsen V, Paponov I, Friml J, Heidstra Renze, Aida M, Palme K, Scheres B. The PIN auxin efflux facilitator network controls growth and patterning in Arabidopsis roots. Nature. 2005;433:39–44. 10.1038/nature03184. 15635403

[25] Xu M, Zhu L, Shou HX, Wu P. A PIN1 family gene, OsPIN1, involved in auxin-dependent adventitious root emergence and tillering in rice. Plant Cell Physiol. 2005;46:1674–81. 10.1093/pcp/pci183. 16085936

[26] Sun HW, Guo XL, Xu FG, Wu DX, Zhang XH, Lou MM, Luo FF, Xu GH, Zhang YL. Overexpression of OsPIN2 regulates root growth and function in response to phosphate deficiency in rice. Int J Mol Sci. 2019;20:5144. 10.3390/ijms20205144. 31627334 PMC6829224

[27] Gutiérrez RA, Lejay LV, Dean A, Chiaromonte F, Shasha D, Coruzzi GM. Qualitative network models and genome-wide expression data define carbon/ nitrogen-responsive molecular machines in Arabidopsis. Genome Biol. 2007;8:1–13. http://genomebiology.com/2007/8/1/R7. 10.1186/gb-2007-8-1-r7 PMC1839130 17217541

[28] Vidal EA, Araus V, Lu C, Parry G, Green PJ, Coruzzi GM, Gutiérrez RA. Nitrate-responsive miR393/AFB3 regulatory module controls root system architecture in Arabidopsis thaliana. Proc Natl Acad Sci USA. 2010;107:4477–82. 10.1073/pnas.0909571107. 20142497 PMC2840086

[29] Vega A, Fredes I, O’Brien J, Shen ZX, Ötvös K, Abualia R, Benkova E, Briggs SP, Gutierrez RA. Nitrate triggered phosphoproteome changes and a PIN2 phosphosite modulating root system architecture. EMBO Rep. 2021;22:e51813. 10.15252/embr.202051813. 34357701 PMC8447600

[30] Song WJ, Sun HW, Li J, Gong XP, Huang SJ, Zhu XD, Zhang YL, Xu GH. Auxin distribution is differentially affected by nitrate in roots of two rice cultivars differing in responsiveness to nitrogen. Ann Bot. 2013;112:1383–93. 10.1093/aob/mct212. 24095838 PMC3806541

[31] Wang BB, Zhu XL, Guo XL, Qi XJ, Feng F, Zhang YL, Zhao QZ, Han D, Sun HW. Nitrate Modulates Lateral Root Formation by Regulating the Auxin Response and Transport in Rice. Genes. 2021;12:850. 10.3390/genes12060850. 34205855 PMC8229813

[32] Rogers ED, Benfey PN. Regulation of plant root system architecture: implications for crop advancement. Curr Opin Biotechnol. 2015;32:93–8. 10.1016/j.copbio.2014.11.015. 25448235

[33] Raun WR, Johnson GV. Improving nitrogen use efficiency for cereal production. Agron J. 1999;91:357–63. 10.2134/agronj1999.00021962009100030001x.

[34] Li ZX, Zhang XR, Zhao YJ, Li YJ, Zhang GF, Peng ZH, Zhang JR. Enhancing auxin accumulation in maize root tip improves root growth and dwarfs plant height. Plant Biotechnol J. 2017;16:86–99. 10.1111/pbi.12751. 28499064 PMC5785362

[35] Liu J, Hasanuzzaman M, Sun HZ, Zhang J, Peng T, Sun HW, Xin ZY, Zhao QZ. Comparative morphological and transcriptomic responses of lowland and upland rice to root-zone hypoxia. Environ Exp Bot. 2019;169:103916. 10.1016/j.envexpbot.2019.103916.

[36] Hu WJ, Chen L, Qiu XY, Wei J, Lu HL, Sun GC, Ma XF, Yang ZR, Zhu CQ, Hou YQ, Shen GX. AKR2A participates in the regulation of cotton fibre development by modulating biosynthesis of very-long-chain fatty acids. Plant Biotechnol J. 2020;18:526–39. 10.1111/pbi.13221. 31350932 PMC6953204

[37] Liu XW, Lin YM, Liu DQ, Wang CX, Zhao ZQ, Cui XM, Liu Y, Yang Y. MAPK-mediated auxin signal transduction pathways regulate the malic acid secretion under aluminum stress in wheat (Triticum aestivum L.). Sci Rep. 2017;7:1620. 10.1038/s41598-017-01803-3. 28487539 PMC5431644

[38] Zhang ML, Lu XD, Li CL, Zhang B, Zhang CY, Zhang XS, Ding ZJ. Auxin efflux carrier ZmPGP1 mediates root growth. Plant Physiol. 2018;177:819–32. 10.1104/pp.17.01379. 29720555 PMC6001327

[39] Okushima Y, Fukaki H, Onoda M, Theologis A, Tasaka M. ARF7 and ARF19 regulate lateral root formation via direct activation of LBD/ASL genes in Arabidopsis. Plant Cell. 2007;19:118–30. 10.1105/tpc.106.047761. 17259263 PMC1820965

[40] Lee HW, Kim NY, Lee DJ, Kim J. LBD18/ASL20 regulates lateral root formation in combination with LBD16/ASL18 downstream of ARF7 and ARF19 in Arabidopsis. Plant Physiol. 2009;151:1377–89. 10.1104/pp.109.143685. 19717544 PMC2773067

[41] Lee HW, Cho C, Kim J. Lateral organ boundaries domain16 and 18 Act downstream of the AUXIN1 and LIKE-AUXIN3 auxin influx carriers to control lateral root development in Arabidopsis. Plant Physiol. 2015;168:1792–806. 10.1104/pp.15.00578. 26059335 PMC4528759

[42] Hu SK, Zhang M, Yang YQ, Xuan W, Zou ZW, Arkorful E, Chen Y, Ma QP, Anburaj J, Chen X, Li XH. A novel insight into nitrogen and auxin signaling in lateral root formation in tea plant [Camellia sinensis (L.) O. Kuntze]. BMC Plant Biol. 2020;20:232. 10.1186/s12870-020-02448-7. 32448156 PMC7247184

[43] Vanneste S, Friml J. Auxin: A trigger for change in plant development. Cell. 2009;136:1005–16. 19303845 10.1016/j.cell.2009.03.001

[44] Zhang XR, Wang BM, Zhao YJ, Zhang JR, Li ZX. Auxin and GA signaling play important roles in the maize response to phosphate deficiency. Plant Sci. 2019;283:177–88. 10.1016/j.plantsci.2019.02.011. 31128687

[45] Gallavotti A, Yang Y, Schmidt RJ, Jackson D. The relationship between auxin transport and maize branching. Plant Physiol. 2008;147:1913–23. 10.1104/pp.108.121541. 18550681 PMC2492655

[46] Pan XQ, Welti R, Wang XM. Quantitative analysis of major plant hormones in crude plant extracts by high-performance liquid chromatography-mass spectrometry. Nat Protoc. 2010;5:986–92. 10.1038/nprot.2010.37. 20448544

[47] Livak KJ, Schmittgen TD. Analysis of relative gene expression data using real-time quantitative PCR and the 2-^ΔΔCT^ method. Methods. 2001;25:402–8. 10.1006/meth.2001.1262. 11846609

[48] Juarez MT, Kui JS, Thoas J, Heller BA, Timmermans MCP. microRNA-mediated repression of rolled leaf1 specifies maize leaf polarity. Nature. 2004;428:84–8. 10.1038/nature02363. 14999285

[49] Sun X, Chen H, Wang P, Chen FJ, Yuan L, Mi G. Low nitrogen induces root elongation via auxin-induced acid growth and auxin-regulated target of rapamycin (TOR) pathway in maize. J Plant Physiology. 2020;254:153281. 10.1111/j.1365-3040.2005.01393.x. 32971423

