# Correction: Auxin efflux carrier *ZmPIN1a* modulates auxin reallocation involved in nitrate-mediated root formation

**DOI:** 10.1186/s12870-026-09971-z

**Published:** 2026-09-21

**Authors:** Yubin Wang, Jiapeng Xing, Jiachi Wan, Qingqing Yao, Yushi Zhang, Guohua Mi, Limei Chen, Zhaohu Li, Mingcai Zhang

**Affiliations:** 1 State Key Laboratory of Plant Physiology and Biochemistry, Collegeof Agronomy and Biotechnology, China Agricultural University, Beijing, 100193 China; 2 Shandong Academy of Agricultural Sciences, Jinan, Shandong China; 3 College of Resources and Environmental Science, China Agricultural University, Beijing, 100193 China; 4 Center for Crop Functional Genomics and Molecular Breeding, China Agricultural University, Beijing, 100193 China

**Correction: BMC Plant Biol 23, 74 (2023)**

**https://doi.org/10.1186/s12870-023-04087-0**

Following publication of the original article [1], the author identified an error in Fig. 5C of the article. The last images in Fig. 5C and D were inadvertently duplicated due to incorrect pasting during the figure preparation process.

The incorrect and the correct figure is given below.

The incorrect Fig. 5 was

**Fig. 5:**
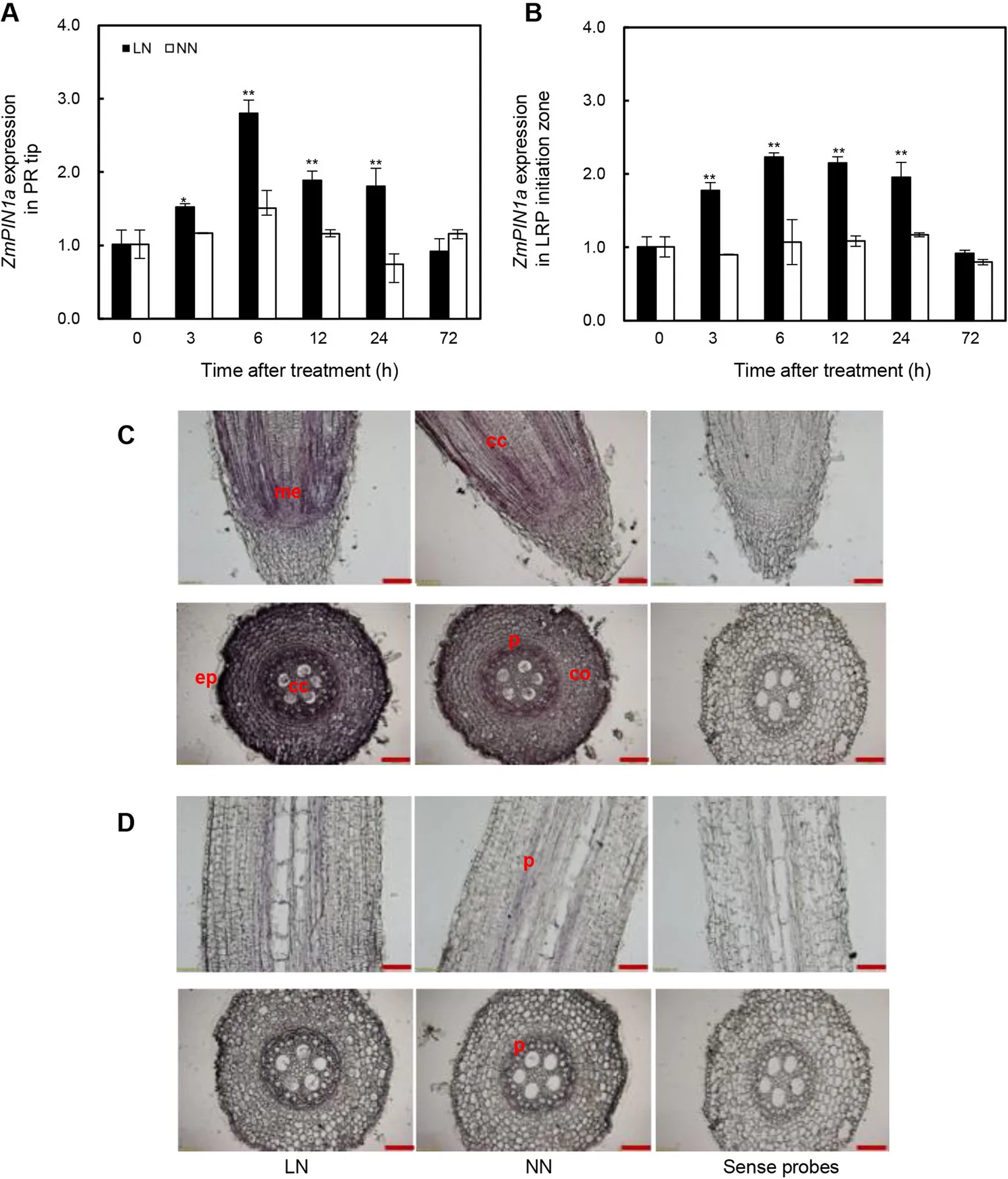
Effect of LN supply on the relative expression of *ZmPIN1a* in roots. **A**-**B** The relative expression of *ZmPIN1a* in PR tip (5 mm of the root tip) (**A**) and LRP initiation zone (5–25 mm behind the root tip) (**B**) at different time points after LN and NN treatments. Values with error bars represent mean ± SD (*n* = 3). The gene expression was calibrated to the expression of *ZmUBC* (ubiquitin C). **C**-**D** In-situ hybridization of *ZmPIN1a* in root at 12 h after LN and NN treatment. Longitudinal and transverse sections of maize PR tip (0–2 mm of the root tip) (**C**) and LRP initiation zone (7–10 mm behind the root tip) (**D**) probed with the *ZmPIN1a* antisense or sense probes. Bar = 100 μm. The asterisks indicated significant differences between different treatments according to a Student’s t test. **, *P* ≤ 0.01

The correct Fig. 5 is

**Fig. 5:**
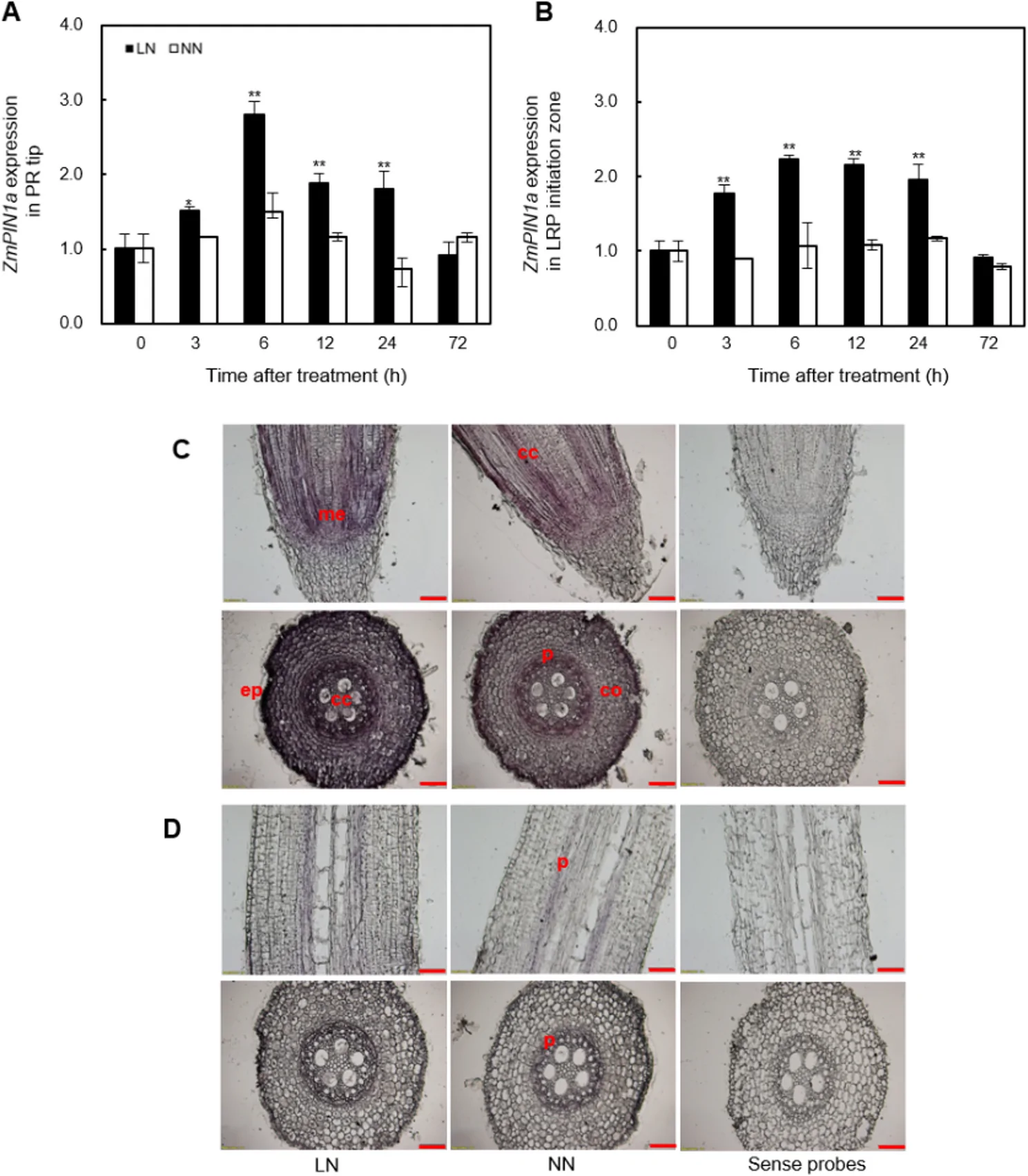
Effect of LN supply on the relative expression of *ZmPIN1a* in roots. **A**-**B** The relative expression of *ZmPIN1a* in PR tip (5 mm of the root tip) (**A**) and LRP initiation zone (5–25 mm behind the root tip) (**B**) at different time points after LN and NN treatments. Values with error bars represent mean ± SD (*n* = 3). The gene expression was calibrated to the expression of *ZmUBC* (ubiquitin C). **C**-**D** In-situ hybridization of *ZmPIN1a* in root at 12 h after LN and NN treatment. Longitudinal and transverse sections of maize PR tip (0–2 mm of the root tip) (**C**) and LRP initiation zone (7–10 mm behind the root tip) (**D**) probed with the *ZmPIN1a* antisense or sense probes. Bar = 100 μm. The asterisks indicated significant differences between different treatments according to a Student’s t test. **, *P* ≤ 0.01

The original article has been updated.
