# Supplementary material for: Auxin efflux carrier ZmPIN1a modulates auxin reallocation involved in nitrate-mediated root formation
Source: BMC Plant Biol. 2023 Feb 3;23:74. doi: 10.1186/s12870-023-04087-0 (PMC9896688; doi:10.1186/s12870-023-04087-0)
Supplement: Supplementary file 1 — Additional file 1:Fig. S1. Effects of auxin on root growth under LN and NN conditions. Fig. S2. Effects of auxin inhibitor NPA on root growth under LN and NN conditions. Fig. S3. Relative expression level of ZmPIN1a in roots of ZmPIN1a-OE plants. Table S1. Effects of LN supply on total root length. Table S2. List of primers used in this study. [file 12870_2023_4087_MOESM1_ESM.docx]

**Supporting information**

**Fig. S1** Effects of auxin on root growth under LN and NN conditions.

**Fig. S2** Effects of auxin inhibitor NPA on root growth under LN and NN conditions.

**Fig. S3** Relative expression level of *ZmPIN1a* in roots of *ZmPIN1a*-OE plants.

**Table S1.** Effects of LN supply on total root length.

**Table S2.** List of primers used in this study.


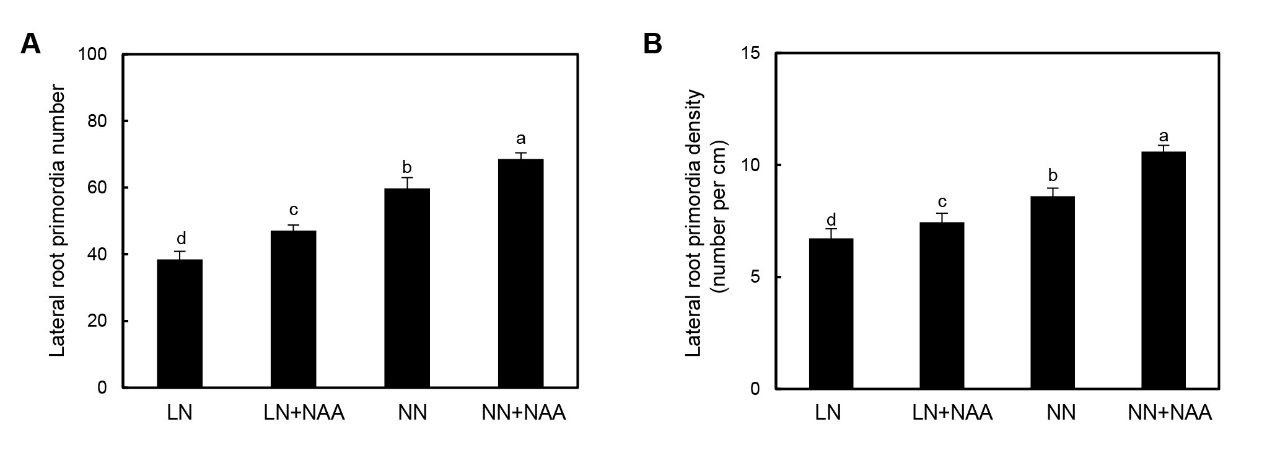


**Fig. S1. Effects of auxin on root growth under LN and NN conditions.**

(A-B) LRP number (A) and density (B) of maize ZD958 in LN and NN solutions containing 1 μM NAA for 2 d. Values with error bars represent mean ± SD (n = 8). Different letters indicated a significant difference between different treatments calculated by Fisher's LSD (P ≤ 0.05). LRP, lateral root primordia; LN, low NO_3_^-^; NN, normal NO_3_^-^.


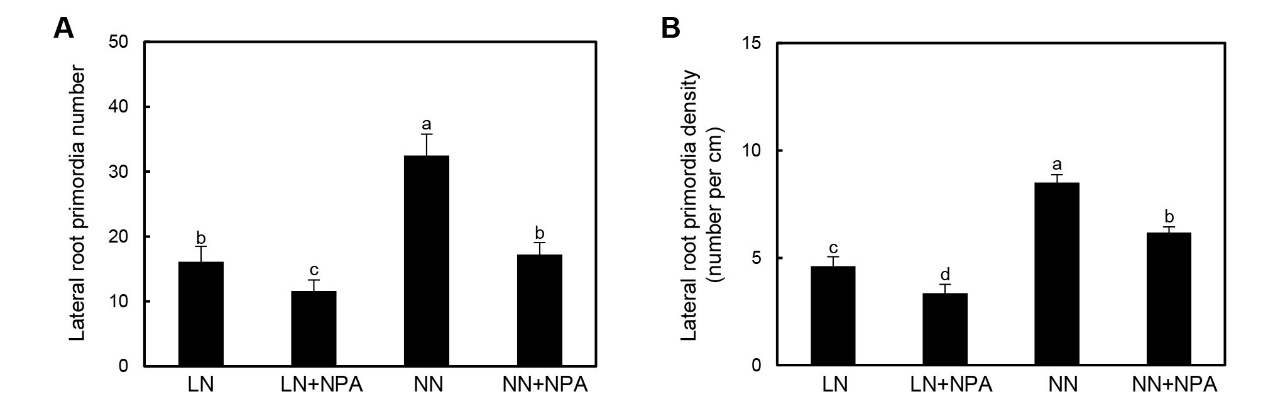


**Fig. S2. Effects of auxin inhibitor NPA on root growth under LN and NN conditions.**

(A-B) LRP number (A) and density (B) of maize ZD958 seedlings in LN and NN solutions containing 0.1 μM NPA for 5 d. Values with error bars represent mean ± SD (n = 8). Different letters indicated a significant difference between different treatments calculated by Fisher's LSD (P ≤ 0.05). LRP, lateral root primordia; LN, low NO_3_^-^; NN, normal NO_3_^-^.


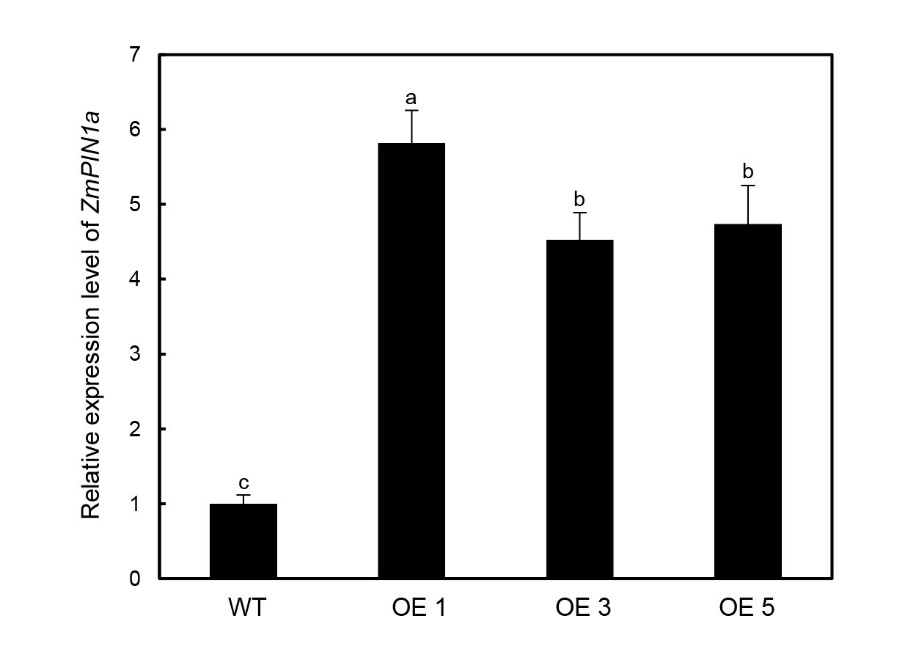


**Fig.S3.** **Relative expression level of *ZmPIN1a* in roots of *ZmPIN1a*-OE plants.**

Roots were harvested at 2 d after cultured in NN solution for qRT-PCR analysis. Values with error bars represent mean ± SD (n = 3). Different letters indicated a significant difference between wild-type and ZmPIN1a-OE plants calculated by Fisher's LSD (P ≤ 0.05). WT, wild-type; NN, normal NO_3_^-^.

**Table S1. Effects of LN supply on total root length**

| **Time (d)** | **treatment** | **Total length of root (cm)** |
| --- | --- | --- |
| 3 | LN | 740 b |
|  | NN | 814 a |
| 5 | LN | 880 b |
|  | NN | 1155 a |

**Table S2. List of primers used in this study**

|  | **Gene name** | | **Sequence (5’-3’)** | | **Sequence (5’-3’)** |
| --- | --- | --- | --- | --- | --- |
| **qRT-PCR primers** | *ZmARF7* | TGGGCAGTCTCGTCGTATAC | | GAAGGCAGAGATGGGTAGC | |
|  | *ZmARF19* | TGTTACTTTGCACGCTGACC | | TCTGTGGCCTTGCATGTTTC | |
|  | *ZmLBD29* | CGGAGTCGGACTACTTGCA | | GAAGTCGAAGGTGTTCCCC | |
|  | *ZmIAA2* | TGAAGGTCAGCATGGACGGC | | TCGCCATCCTTGTCTTCGTA | |
|  | *ZmIAA10* | CAAAGTGGACCTCAAGATGTAC | | TCCTTGTCCTCGTAGGTCGG | |
|  | *ZmIAA21* | CAAGAAGATGTTCGGCACCT | | ATCAACCAATCTCCCCTCGT | |
|  | *ZmPIN1a* | CGGATAATCGCGTGCGGGAACA | | CCGAAGATGACTGCCGTGCTGA | |
|  | *ZmUbiquitin1* | CTGGTGCCCTCTCTCCATATGG | | CAACACTGACACGACTCATGACA | |
| **In suit primers** | *ZmPIN1a-T7* | GATTTAGGTGACACTATAGAATGCTTGCATCATCTGGTACACGCTGAT | | | |
|  | *ZmPIN1a-SP6* | TGTAATACGACTCACTATAGGGTCCACACGAACATGTGTAGGTCCT | | | |
